# Buffering Impostor Feelings with Kindness: The Mediating Role of Self-compassion between Gender-Role Orientation and the Impostor Phenomenon

**DOI:** 10.3389/fpsyg.2017.01289

**Published:** 2017-07-26

**Authors:** Alexandra Patzak, Marlene Kollmayer, Barbara Schober

**Affiliations:** ^1^Faculty of Education, Simon Fraser University, Burnaby BC, Canada; ^2^Department of Applied Psychology: Work, Education and Economy, Faculty of Psychology, University of Vienna Vienna, Austria

**Keywords:** impostor phenomenon, self-compassion, gender-role orientation, gender differences, academic achievement

## Abstract

The impostor phenomenon (IP) refers to high-achievers who underestimate their abilities and thus fear being unmasked as impostors. IP sufferers attribute their success to factors other than their abilities, entailing negative emotions, unfavorable motivations, and reduced well-being. The IP was originally conceptualized as a predominantly female experience, and is thus seen as an important psychological barrier for female academic careers. Empirical findings of gender differences in the IP are equivocal, but sparse research on associations between gender-role orientation and the IP indicates that feminine students suffer more intensely from the IP than masculine students. Femininity and masculinity are also related to self-compassion, a rather young construct that enhances emotional resilience, well-being, and academic achievement. Self-compassion involves being kind to oneself when failing, perceiving one’s inadequacies as part of the human condition, and being mindful about negative aspects of oneself. It reduces fear of failure, denial of competences, and self-doubts which are central components of the IP. However, relations between self-compassion and the IP have not been investigated to date. In this study, we examine self-compassion as a potential resilience factor against the IP, taking gender and gender-role orientation into account. In a cross-sectional online survey, we investigated 459 (315 female) high-achieving first-year undergraduate students. Results include: Female, feminine, and undifferentiated students score higher on measures of the IP and lower on measures of self-compassion than male, masculine, or androgynous students. Higher levels of the IP are associated with lower levels of self-compassion across all students tested. Self-compassion further mediates the relationship between gender-role orientation and the IP. Interventions to enhance self-compassion might thus be an effective way to overcome impostor feelings. Female, feminine, and undifferentiated students might benefit most from facilitation of self-compassion in education.

## Introduction

Women are underrepresented in academic careers across Europe, although the majority of students entering undergraduate programs is female (European Commission, 2016). Various structural and psychological factors, including disadvantageous self-concepts, interests, and motivational and emotional patterns found in women, are associated with this “leaky pipeline” (for an overview see Krais, 2002; Blickenstaff, 2005). In our study, we examine gender differences in the impostor phenomenon (IP) which integrates several usually isolated factors assumed to affect female academic careers to provide explanations for the leaky pipeline, and suggest the rather young construct of self-compassion as a potential resilience factor against the IP.

### The Impostor Phenomenon – a Psychological Barrier to Female Academic Careers

The IP was first conceptualized in the clinical field, where Clance and Imes (1978) worked with highly successful women who held prestigious PhDs, were respected experts in their fields, or excellent students – but nevertheless constantly feared getting unmasked as intellectual impostors. Clance (1985a) described six components characterizing the IP: (1) The Impostor Cycle is a vicious circle of unfavorable learning and attribution styles. To deal with challenges, individuals experiencing the IP either procrastinate and balance that by excessive preparation or immediately prepare excessively. When they are successful with these learning styles, they attribute their success to luck or effort. They believe their success is due to those factors rather than their abilities. Consequently, IP sufferers never feel prepared for the next challenge and hence the impostor cycle starts over again. (2) IP sufferers feel an urgent need to be special, i.e., to not only master challenges but to be outstanding. These unrealistically high expectations trigger self-doubts and explain their reduced perception of capability, despite objective evidence suggesting the opposite. (3) Superwoman/Superman aspects indicate that IP sufferers do not only expect themselves to be outstanding, their effort must also be negligible. This accounts for self-doubts and feelings of fraudulence, especially when IP sufferers cannot live up to their unrealistic expectancies. (4) Fear of failure is fostered by IP sufferers’ fear of getting unmasked as unintelligent, resulting in desperate efforts to avoid any kind of failure. (5) Individuals suffering from the IP also typically deny competence and discount praise. They rely on alternative explanations for their success aside from their abilities. (6) Fear of success is grounded in IP sufferers’ doubts about their ability to repeat success and live up to increased expectations of others.

From this description, it is apparent that the IP integrates several usually isolated constructs such as self-concept, attribution, emotion, and achievement motivation that have a strong impact on educational careers. Therefore, in recent years, the IP has been increasingly proposed as an important psychological factor for career development. The IP has been found to reduce career adaptability resources that are positively related to career planning, career exploration, and occupational self-efficacy and negatively related to career decision-making difficulties (Neureiter and Traut-Mattausch, 2017). Previous research points to the IP as a relevant psychological barrier particularly for young academics for several reasons: First, the university context is structured in a way that enhances the IP (McCormick and Barnes, 2008; Klinkhammer and Saul-Soprun, 2009) as it is characterized by many years of evaluation and examination rituals, evoking feelings of deficiency. Due to intense competition, normal crises like writing blocks or motivational problems are rarely communicated and feelings of inadequacy are often kept secret. In addition, constant adaptation to new role expectations coming with new challenges promotes impostor feelings and provides opportunities to get trapped in the impostor cycle. Moreover, the myth of the ingenious scholar with no need for recreational time aligns to superwoman/supermen aspects experienced by IP sufferers and puts impossible demands on young academics (Macha, 1992). Second, the IP is associated with numerous factors that interfere with academic achievement, such as lower self-esteem (Chrisman et al., 1995; Thompson et al., 1998; Sonnak and Towell, 2001), lower research self-efficacy (Jöstl et al., 2012), lower academic self-concept (Leary et al., 2000; Cokley et al., 2015), lower performance expectancies (Cozzarelli and Major, 1990), and self-perceptions of inadequacy and lack of academic preparedness (Craddock et al., 2011). It is further associated with less favorable achievement goals (Kumar and Jagacinski, 2006) as well as higher depression and anxiety (Bernard et al., 2002; McGregor et al., 2008), higher fear of success (Fried-Buchalter, 1997), and higher fear of failure (Thompson et al., 2000). Third, level of faculty rank is negatively related to the IP, indicating that academics suffering from the IP may be likely to drop-out of academia during the early stages of their careers (Topping and Kimmel, 1985). The IP is thus theoretically linked to academic attrition. Central components of the IP such as feelings of intellectual inadequacy, negative emotions, e.g., fear of failure, fear of success, or anxiety, and negative self-perceptions, e.g., self-doubts, self-criticism, or denial of competences are associated with increased drop-out intentions of university students (Szulecka et al., 1987; Langford and Clance, 1993; Bask and Salmela-Aro, 2013; Cortes et al., 2014). This makes especially students suffering from the IP vulnerable to drop-out of their academic programs. The first year at universities is particularly crucial for academic attrition and might be a fertile soil for the IP (e.g., Szulecka et al., 1987; Baars and Arnold, 2014). Adjusting to this unfamiliar learning environment is highly demanding and distressing for first-year undergraduates, especially for those who experience impostor feelings.

From their experiences in clinical treatment, Clance and Imes (1978) conceptualized the IP as a distinctly female experience. They concluded that women are more likely than men to attribute the reason for success outward – either to an external cause (e.g., luck) or a temporary internal quality (e.g., effort) – than to inherent ability. They explained this pattern by internalization of the societal gender stereotype that women are not considered competent (Furnham et al., 2002; Bian et al., 2017). Therefore, high-achieving women must find alternative explanations for their success. However, empirical findings of gender differences in the IP are equivocal: In college and university students, some studies found that women are more affected by the IP than men (King and Cooley, 1995; Kumar and Jagacinski, 2006; McGregor et al., 2008), while others did not find gender differences (Cowman and Ferrari, 2002; Cokley et al., 2013). In faculty members, Jöstl et al. (2012) found higher impostor scores in women, while Topping and Kimmel (1985) found the contrary and Hutchins (2015) found no gender differences. These studies investigated gender differences by simply comparing men and women. However, when examining gender differences, researchers should also consider the extent to which individuals identify with typical characteristics of masculinity and femininity, i.e., their gender-role orientation. Bem (1974) suggests that psychological masculinity and femininity are independent dimensions and that individuals can be high or low on each dimension, irrespective of their gender. Masculinity has been associated with achievement-oriented traits, labeled as agency or instrumentality, whereas femininity has been associated with social-oriented traits, designated as communion or expressivity (Kite et al., 2008). Spence et al. (1975) suggest that androgyny, i.e., a high degree of both, masculinity and femininity, is most desirable as it integrates strength of both dimensions. Possessing few characteristics of femininity and masculinity, i.e., an undifferentiated gender-role orientation, is thus less desirable and associated with less effective functioning compared to the other three types of gender-role orientation. Bem (1974) argues, that the adoption of either gender-role orientation can over-ride one’s gender regarding its impact on psychological functioning. Nevertheless, only limited research has addressed the relationship between gender-role orientation and the IP to date. September et al. (2001) found that students with feminine and undifferentiated gender-role orientation reported higher levels of the IP and lower levels of well-being than those with masculine or androgynous gender-role orientation.

Overall, research supports the assumption that the IP affects academic careers. Imposter experiences cause feelings of intellectual and professional incapability despite objective evidence to the contrary. They entail negative emotions and motivational aspects that interfere with academic achievement and are associated with academic drop-out. Although the IP was originally conceptualized as a distinctly female experience, findings of gender differences in the IP are equivocal and research on gender-role orientation and the IP is sparse. Therefore, we examine gender differences in the IP by focusing on gender and gender-role orientation simultaneously. This approach allows us to investigate the influence of gender and gender-role orientation on the IP, as well as their interaction.

Research Question 1: How are gender and gender-role orientation related to the impostor phenomenon?

### Self-compassion as a Psychological Resilience Factor against the IP

Finding resilience factors and interventions against the IP may contribute to preventing young and promising academics from attrition. In our view, self-compassion – an important Buddhist concept becoming increasingly popular in Western psychology – might be a promising psychological resilience factor against the IP. Psychological research on self-compassion is relatively new, but highly relevant to researchers interested in self-concepts and self-attitudes in relation to well-being. In contrast to self-esteem, which is built on evaluations of self-worth and constituted by judgments and comparisons (Crocker and Park, 2004), self-compassion is related to compassion (Neff, 2003a). Compassion entails being empathetic to the suffering of others, and offering non-judgmental understanding to persons who fail or do wrong, so that their actions and behaviors are seen as part of the larger human experience (Gilbert, 2005). Self-compassion therefore involves being sensitive to one’s own suffering and offering non-judgmental understanding to one’s own pain, inadequacies and failures, so that one’s experience is perceived in the context of shared human fallibility (Neff, 2003a,b). Three basic components constitute self-compassion (Neff, 2003a): (1) Self-kindness, i.e., being kind and understanding to oneself rather than judgmental, (2) common humanity, i.e., recognizing that all humans are imperfect, fail, and make mistakes and seeing one’s experiences as part of the larger human experience instead of separating or isolating, and (3) mindfulness, i.e., being aware of one’s present experience in a balanced manner rather than over-identifying with negative aspects of one’s life. The basic components of self-compassion are conceptually distinct and are experienced differently, but also interact by mutually enhancing and engendering one another. On the one hand, a certain amount of mindfulness is needed to keep enough mental distance from negative experiences to allow feelings of self-kindness and common humanity. On the other hand, mindfulness directly contributes to the other two components by reducing self-criticism and increasing self-understanding, thus enhancing self-kindness and by antagonizing egocentrism, entailing feelings of isolation, thus increasing feelings of interconnectedness and common humanity.

Self-compassion is negatively related to depression, anxiety, and stress (Barnard and Curry, 2012; MacBeth and Gumley, 2012), and facilitates resilience by moderating reactions of individuals to negative events. Moreover, self-compassion is linked to increased motivation, and resilient coping (e.g., Allen et al., 2012; Breines and Chen, 2012; Sbarra et al., 2012). According to Neff et al. (2005), students’ self-compassion is positively associated with mastery goals and negatively associated with performance goals. This relationship is mediated by the greater perceived competence and lesser fear of failure of self-compassionate individuals. In case of perceived academic failure, self-compassion is positively related to emotion-focused coping strategies and negatively related to avoidance-oriented strategies. These motivational aspects associated with self-compassion are advantageous in academic contexts.

Evidence suggesting positive associations between self-compassion and well-being set the stage for the development of several interventions tailored to enhance self-compassion. The general therapeutic approach compassion-focused therapy (CFT), helping clients to develop a self-compassionate mind, especially when suffering from shame and self-attack (Gilbert, 2005), or the mindful self-compassion (MSC) program, which significantly increases self-compassion and life satisfaction while reducing depression, anxiety, and stress (Neff and Germer, 2013) are only two out of numerous self-compassion interventions. Moreover, various self-compassion guided meditations and exercises can be accessed freely online (e.g., Neff, 2017). These low-threshold interventions support individuals in developing a new way of relating to themselves by increasing self-compassion.

High-achievers suffering from the IP – an intense, subjective self-perception of phoniness – believe that their success is due to mistake, and live in constant fear of getting unmasked as intellectual impostors. Therefore, there is a clear theoretical link between the IP and self-compassion. IP sufferers are harshly critical and judgmental with themselves depicted in superman/superwomen aspects, their denial of competence, and discount of praise, i.e., they lack self-kindness. They think that they are different from others which is illustrated by their need to be special. They feel that only they are imperfect, fail, and make mistakes, entailing fear of failure and fear of success, i.e., they lack common humanity. IP sufferers further over-identify with negative aspects of themselves, trapping them in the impostor cycle, i.e., they lack mindfulness. Therefore, self-compassion seems to be a promising resilience factor against the IP that can be enhanced by therapeutic interventions. Even though self-compassion is theoretically related to the IP and appears to be a promising resilience factor for the IP, these constructs have not been linked in research to date. Therefore, we aim to bridge this research gap by examining the relation between self-compassion and the IP.

Research Question 2: How is self-compassion related to the impostor phenomenon?

In our study, we aim to gain new insights into psychological barriers for female university careers by focusing on gender, the IP, and self-compassion in first-year undergraduate students. Theoretical considerations suggest negative associations between self-compassion and the IP. Building on equivocal findings of gender differences in the IP (Kumar and Jagacinski, 2006; McGregor et al., 2008; Jöstl et al., 2012), we apply a broader approach and take gender as well as gender-role orientation into account. Empirical research indicates positive associations between feminine gender-role orientation and the IP (September et al., 2001). Gender influences not only the IP but also self-compassion. Males were found to show higher levels of self-compassion than females (Neff et al., 2008; Neff and McGehee, 2010; Yarnell et al., 2015). Research addressing the relationship between self-compassion and gender-role orientation is sparse. Neff (2003b) has indicated that individuals with higher levels of self-compassion are more likely to report that they are equally kind toward themselves as they are to others, whereas individuals with lower levels of self-compassion are more likely to report an imbalance in this context. Therefore, women and men who identify strongly with either masculinity or femininity, are likely to experience low levels of self-compassion for different reasons. Individuals with feminine gender-role orientation prioritize relationships over the self, while individuals with masculine gender-role orientation prioritize the self over relationships. Tatum (2013), however, found positive associations of masculinity and femininity with self-compassion, with masculine gender-role orientation accounting for the greatest proportion of variance in self-compassion. Therefore, the relationship between gender-role orientation and the IP might be mediated by self-compassion. Arguing that self-compassion might be a resilience factor against the IP, we assume that self-compassion mitigates the IP, and consequently reduces the strength of the relationship between gender-role orientation and the IP.

Research Question 3: Is the relationship between gender-role orientation and the IP mediated by self-compassion?

## Materials and Methods

### Participants and Procedure

An email invitation to participate in this study was sent to all first-year undergraduates enrolled at four of the five largest universities in Vienna, Austria. We focused on undergraduate students in their first year as the IP is not well researched in this sample, even though impostor experiences are theoretically linked to drop-out intentions and first-year undergraduates are especially vulnerable to academic attrition (e.g., Clance, 1985b; Szulecka et al., 1987; Baars and Arnold, 2014). In this early stage of academic careers, we thus expect that IP experiences begin to unfold which offers opportunities for early interventions. 945 students volunteered to participate in this study, of which 72% completely answered the questionnaire. As high achievement is a prerequisite for the IP, exclusively high-achievers (GPA > 3.0 in the school leaving examination) were included in the final sample (*n* = 459). Participants’ ages ranged from 17 to 65 years (*M* = 21, *SD* = 5.60) and 69% of participants were female, which is representative for this population (Bundesministerium für Wissenschaft, Forschung und Wirtschaft [BMWFW], 2017).

The present study was designed as a cross-sectional online survey. An online consent form informed participants about duration, procedure, and goals of this study. Participants were guaranteed anonymity and confidentiality of their data and were informed that participation was voluntary and could be withdrawn at any point of the questionnaire. After completing the informed consent form, participants answered an online questionnaire spanning demographics (gender, age, and affiliation), GPA, and measures of the IP, gender-role orientation, and self-compassion.

### Material and Measures

To minimize bias, measures of the IP, gender-role orientation, and self-compassion as well as corresponding items implemented in the questionnaire were presented in random order. We used a 5-point rating scale ranging from 0 = not at all true to 4 = very true for all measures.

### Impostor Phenomenon

The IP was assessed using the German version of the Clance Impostor Phenomenon Scale (CIPS; Klinkhammer and Saul-Soprun, 2009), a 20-item scale (Cronbach α = 0.90) representing components of the IP (sample item: “Sometimes I’m afraid others will discover how much knowledge or ability I really lack”). Higher scores indicate higher levels of the IP. Impostor experiences were grouped into four levels (few, moderate, frequent, and intense) based on the magnitude of the overall score applying the cut-off scores proposed by Clance (1985b).

### Gender-Role Orientation

Gender-role orientation was measured using a short version of the Bem Sex Role Inventory (BSRI; Bem, 1974) in German language, based on Schertzer et al. (2008). A confirmatory factor analysis (CFA) of this 12-item scale was conducted. The two factors represent the subscales feminine and masculine gender-role orientation and fit the data reasonably well [χ^2^(53) = 271.76, *p* < 0.001], see **Table 1** for factor loadings. Factor loadings were transformed to the same scale by dividing feminine and masculine loadings by their sums. Cronbach’s α of the subscales was α_fem_ = 0.78 and α_mas_ = 0.75. Sample items are “I am understanding” (femininity) and “I am ambitious” (masculinity). Using the median-split method (Spence and Helmreich, 1972), four groups of gender-role orientations were obtained as illustrated in **Table 2**: feminine, masculine, androgynous, and undifferentiated gender-role orientation.

**Table 1:** Factor loadings of the measure of gender-role orientation.

	Loadings on		Loadings on
Item	femininity	Item	masculinity
I am understanding	1.00	I am dominant	1.00
I am compassionate	1.36	I am ambitious	0.52
I am affectionate	1.44	I am assertive	1.05
I am gentle	-0.07	I am competitive	0.54
I am sensitive to the needs of others	1.05	I have leadership abilities	1.09
I am warm	1.21	I am willing to take a stand	0.81

*Factor loadings are unstandardized.*

**Table 2:** Illustration on how to obtain gender-role orientations when applying the median-split method (Spence and Helmreich, 1972).

		Subscale feminine	
		Low	High
Subscale masculine	Low	Undifferentiated	Feminine
	High	Masculine	Androgynous

*Low = scores smaller than the median, high = scores greater than the median.*

### Self-compassion

Hupfeld and Ruffieux (2011) German version of the Self-Compassion Scale (SCS-D) was used to assess self-compassion. SCS-D is a 26-item scale (Cronbach α = 0.89) including six subscales that contrast positive and negative characteristics of the three components of self-compassion: self-kindness (sample item “I try to be understanding and patient toward aspects of my personality I don’t like”) vs. self-judgment (sample item “When I see aspects of myself that I don’t like, I get down on myself”’), common humanity (sample item “I try to see my failings as part of the human condition”) vs. isolation (sample item “When I fail at something that’s important to me, I tend to feel alone in my failure”), mindfulness (sample item “When something upsets me I try to keep my emotions in balance”) vs. over-identification (sample item “When something painful happens I tend to blow the incident out of proportion”). Internal consistencies for the subscales were good, with α_sk_ = 0.88, α_sj_ = 0.88, α_ch_ = 0.89, α_i_ = 0.88, α_m_ = 0.88, and α_oi_ = 0.88. Items from subscales addressing negative characteristics were reverse coded so that higher scores indicate higher levels of self-compassion.

## Results

Data was analyzed using the software environment for statistical computing R (R Core Team, 2017). To answer the research questions, we conducted the following statistical tests: ANOVA, *t*-tests, ordered logistic regression, and path analyses. Effect sizes were computed and a significance level of 0.05 was used throughout this study.

### Gender Differences in the Impostor Phenomenon

We conducted an ANOVA to examine differences in the IP between individuals of different gender and gender-role orientation, see **Table 3** for the distribution of types of gender-role orientation in male and female participants. Gender with the categories female and male and gender-role orientation with the categories undifferentiated, feminine, masculine, and androgynous were included as independent variables. Results indicate that gender and gender-role orientation were both statistically detectably associated with the IP [*F*(1,451)_gender_ = 7.54, *p* = 0.006, ηg_gender_^2^ = 0.02; *F*(3,451)_GRO_ = 6.38, *p* < 0.001, ηg_GRO_^2^ = 0.04]. No statistically detectable interaction effect was found [*F*(3,451)_GRO^∗^gender_ = 0.36, *p* = 0.76]. Results indicate that male students suffered less intensely from the IP than female students, see **Table 4**. The relationship between gender-role orientation and the IP is illustrated in **Table 5**. Masculine (*M* = 34.3, *SD* = 15.5) and androgynous students (*M* = 36.8, *SD* = 16.4) suffered less intensely from the IP than feminine (*M* = 39.8, *SD* = 14.1) or undifferentiated students (*M* = 42.6, *SD* = 15.4). *Post hoc* pairwise *t*-tests with Bonferroni corrected *p*-values revealed statistically detectable mean differences between participants with masculine and feminine gender-role orientation (*p* = 0.03), between participants with masculine and undifferentiated gender-role orientation (*p* < 0.001), as well as between participants with androgynous and undifferentiated gender-role orientation (*p* = 0.03). No statistically detectable differences in the IP were found between students with feminine and undifferentiated gender-role orientation as well as between students with masculine and androgynous gender-role orientation (*p* > 0.05). This indicates that four categories of gender-role orientation are too differentiated. We thus used the scores on the two factors femininity and masculinity specified by CFA (reported above) for further analyses.

**Table 3:** Distribution of gender-role orientations in male and female participants.

	Gender-role orientation			
	Undifferentiated	Feminine	Masculine	Androgynous
Total (%)	110 (24%)	120 (26%)	120 (26%)	109 (24%)
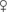 students (%)	75 (17%)	93 (30%)	66 (21%)	81 (26%)
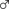 students (%)	55 (24%)	27 (19%)	54 (38%)	28 (19%)

**n* = 459 students, *n*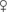 = 315 female students, *n*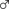 = 144 male students.*

**Table 4:** Distribution of impostor feelings by male and female participants.

	Impostor experiences			
	Few	Moderate	Frequent	Intense
Total (%)	62 (14%)	213 (46%)	142 (31%)	42 (9%)
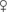 students (%)	37 (12%)	142 (45%)	100 (32%)	36 (11%)
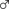 students (%)	25 (18%)	71 (49%)	42 (29%)	6 (4%)

**n* = 459 students, *n*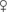 = 315 female students, *n*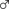 = 144 male students.*

**Table 5:** Distribution of impostor feelings across gender-role orientation.

	Impostor experiences			
	Few	Moderate	Frequent	Intense
Undifferentiated (%)	9 (8%)	48 (44%)	36 (33%)	17 (15%)
Feminine (%)	8 (7%)	60 (50%)	44 (36%)	8 (7%)
Masculine (%)	26 (21%)	55 (46%)	31 (26%)	8 (7%)
Androgynous (%)	19 (17%)	50 (46%)	31 (29%)	9 (8%)

**n* = 459, *n*_*undifferentiated*_ = 110, *n*_*feminine*_ = 120, *n*_*masculine*_ = 120, *n*_*androgynous*_ = 109.*

### Self-compassion and the Impostor Phenomenon

To analyze the relationship between self-compassion and the IP, Pearson’s product moment correlation, Welch’s *t*-tests, and ordered logistic regression were conducted.

**FIGURE 1:**
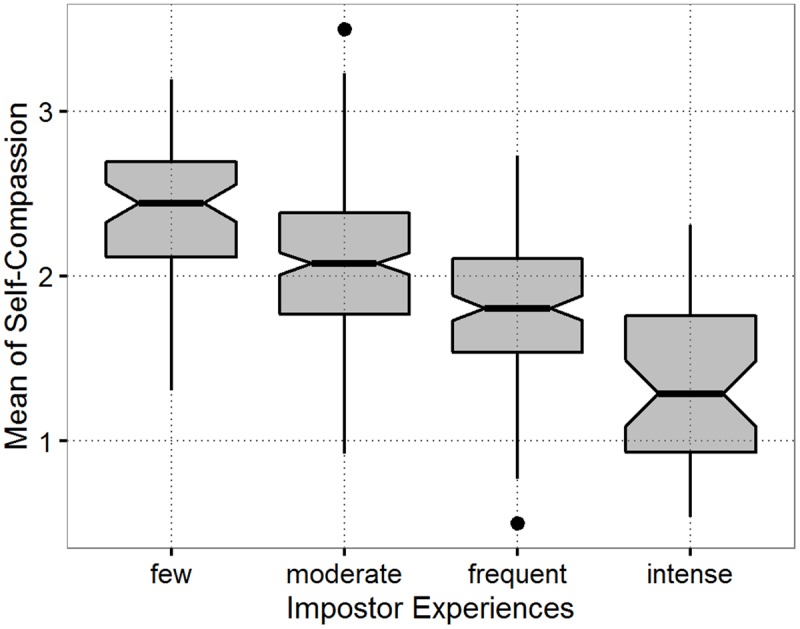
Distribution of mean values of self-compassion by intensity levels of impostor experiences.

Pearson’s product moment correlation indicates that self-compassion is negatively correlated to the IP with a large effect size (*r* = -0.55, *p* < 0.001). Kolmogorov–Smirnov tests of the mean of self-compassion in each level of impostor experiences indicate that the requirement for the *t*-test of normal distribution in each group is met (*D*_few_ = 0.08, *p* = 0.83; *D*_moderate_ = 0.04, *p* = 0.96; *D*_frequent_ = 0.05, *p* = 0.82; *D*_intense_ = 0.11, *p* = 0.72). Conducting *t*-tests is thus a sensible choice. Welch’s *t*-tests were used to test for mean differences in self-compassion between every two consecutive levels of IP experiences, accounting for unequal variances. In doing so, three *t*-tests with Bonferroni correction were conducted, see **Table 6** for results. Self-compassion differed statistically detectably between the four IP intensity levels with medium to large effect sizes. With increasing intensity of the IP, self-compassion decreases, see boxplots in **Figure 1**.

**Table 6:** Mean differences of self-compassion between two consecutive levels of intensity of impostor experiences.

	*Df*	*t*	Cohen’s *d*	*p*-value
Few^∗^Moderate	106.81	4.98	0.69	0.0001
Moderate^∗^Frequent	311.37	6.21	0.67	0.0001
Frequent^∗^Intense	60.42	5.28	1.00	0.0001

Ordered logistic regression was conducted with self-compassion as independent variable and the IP with four ordered categories (few, moderate, frequent, and intense) as the dependent variable. The resulting odds ration OR = 0.11 fell in the 95% confidence interval CI95% [0.07,0.16] and is thus statistically detectable. Results indicate that the likelihood of high self-compassion to occur in high levels of the IP is 1/10.

Additionally, Pearson product moment correlations of the self-compassion subscales revealed medium negative correlations between the IP and self-kindness (*r*_sk_ = -0.29, *p* < 0.001) as well as mindfulness (*r*_m_ = -0.18, *p* < 0.001). The negative subscales self-judgment, over-identification, and isolation were highly positively correlated with the IP (*r*_sj_ = 0.51, *p* < 0.001; *r*_oi_ = 0.53, *p* < 0.001; *r*_i_ = 0.69, *p* < 0.001). Common humanity was the only subscale without a statistically detectable correlation with the IP (*r*_ch_ = 0.09, *p* = 0.07).

### Gender-Role Orientation, Self-compassion, and the Impostor Phenomenon

Path analysis was conducted with the R package sem (Fox et al., 2015) to examine our theoretical model spanning gender-role orientation, self-compassion, and the IP. We hypothesized that the relationship between feminine and masculine gender-role orientation (independent variables) and the IP (dependent variable) is mediated by self-compassion.

**FIGURE 2:**
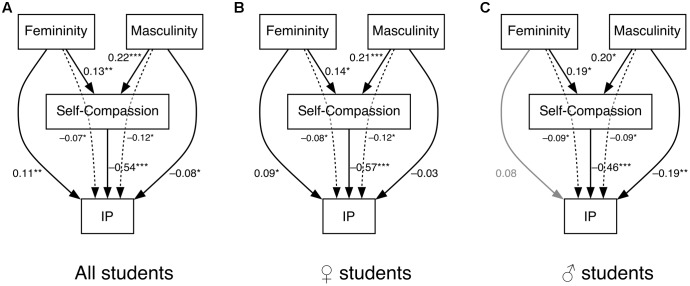
Path model of gender-role orientation (i.e., feminine, masculine), self-compassion, and the impostor phenomenon (IP) for **(A)** all students, **(B)** exclusively female students, and **(C)** exclusively male students. Solid lines represent direct effects, dotted lines represent indirect effects, lighter lines represent non-statistically detectable effects, ^∗^*p* < 0.05, ^∗∗^*p* < 0.01, ^∗∗∗^*p* < 0.001.

Following Zhao et al. (2010) recommendation, we estimated 95% confidence intervals using bootstrapping in addition to the point estimates obtained by path analysis. Three path analyses were conducted for (a) the entire sample (*n* = 459), (b) exclusively female students (*n* = 315), and (c) exclusively male students (*n* = 144). The model fits the data reasonably well for all samples [χ^2^(1,459)_a_ = 0.04, *p* = 0.84; χ^2^(1,315)_b_ = 0.84, *p* = 0.36; χ^2^(1,144)_c_ = 0.71, *p* = 0.40]. Results are presented in **Figure 2**. For an overview of mean values of the IP and self-compassion among gender and gender-role orientation see **Table 7**.

**Table 7:** Mean values of the impostor phenomenon and self-compassion among gender and gender-role orientation.

	Gender		Gender-role orientation			
	Female	Male	Undiff.	Feminine	Masc.	And.
Self-compassion	1.91	2.07	1.81	1.92	2.00	2.11
Impostor						
phenomenon	1.99	1.75	2.13	1.99	1.72	1.84

*IP and self-compassion were measured on a 5-point scale, ranging from 0 to 4; undiff. = undifferentiated, mas. = masculine, and. = androgynous.*

When all participants were included in the model, all effects were statistically detectable. Feminine and masculine gender-role orientation was positively associated with self-compassion whereas masculine gender-role orientation was associated with self-compassion to a greater extent (*de*_fem__×__SC_ = 0.13, *p* = 0.006; *de*_mas__×__SC_ = 0.22, *p* < 0.001). Self-compassion was further highly negatively associated with the IP (*de*_SC__×__IP_ = -0.54, *p* < 0.001), confirming previous results. As predicted, feminine gender-role orientation was positively associated with the IP (*de*_fem__×__IP_ = 0.11, *p* = 0.006) whereas masculine gender-role orientation was negatively associated with the IP (*de*_mas__×__IP_ = -0.08, *p* = 0.04). Indirect effects of feminine or masculine gender-role orientation through self-compassion to the IP were statistically detectable (*ie*_fem__×__SC__×__IP_ = -0.07, CI95% [-0.01; -0.13]; *ie*_mas__×__SC__×__IP_ = -0.12, CI95% [-0.06; -0.19]), indicating that self-compassion mediates between gender-role orientation and the IP. Direct and indirect effects exist between gender-role orientation and the IP. Whereas for masculinity both effects are negative (i.e., complementary mediation), direct and indirect effects for femininity point in opposite directions indicating a competitive mediation effect (Zhao et al., 2010).

Applying the model to female and male students separately, effects are similar except for a non-statistically detectable direct effect between masculine gender-role orientation and the IP for female students (*de* = -0.03, *p* = 0.45), and feminine gender-role orientation and the IP for male students (*de* = 0.08, *p* = 0.27). Self-compassion statistically detectably mediated between feminine or masculine gender-role orientation and the IP in all three samples, confirming our hypothesis.

## Discussion

The broader aim of this study was to enhance understanding of the leaky pipeline of female academic careers. Even though female students outnumber males in undergraduate programs, females are underrepresented in academic careers (European Commission, 2016). To gain insights in this gender imbalance, we focused on first-year undergraduates and examined gender differences in the IP as a potential explanatory factor for the leaky pipeline, and self-compassion as a possible resilience factor against the IP.

Research indicates that first-year undergraduate students are especially vulnerable to drop-out of their academic programs (Szulecka et al., 1987; Baars and Arnold, 2014). Academic attrition is theoretically related to the IP, which interferes with academic achievement and hinders academic careers (Topping and Kimmel, 1985; Cozzarelli and Major, 1990; Chrisman et al., 1995; Fried-Buchalter, 1997; Thompson et al., 1998, 2000; Leary et al., 2000; Sonnak and Towell, 2001; Kumar and Jagacinski, 2006; Jöstl et al., 2012; Cokley et al., 2015). Individuals suffering from the IP are high-achievers who have a reduced perception of their abilities and thus feel like they do not deserve their success and are afraid of getting unmasked as unintelligent (Clance, 1985b). These feelings of intellectual fraudulence are associated with negative emotional and motivational aspects, and disadvantages in academic contexts (Fried-Buchalter, 1997; Thompson et al., 2000; Kumar and Jagacinski, 2006; McGregor et al., 2008; Jöstl et al., 2012). The IP was highly present in first-year undergraduate students in this study. 60% of students experienced at least moderate impostor feelings, emphasizing the relevance of the IP for young academics. This is in line with findings that university contexts are catalysts for the IP (McCormick and Barnes, 2008; Klinkhammer and Saul-Soprun, 2009).

Although Clance and Imes (1978) introduced the IP as a predominantly female experience, findings of gender differences in the IP are equivocal (e.g., Topping and Kimmel, 1985; King and Cooley, 1995; Kumar and Jagacinski, 2006; McGregor et al., 2008; Jöstl et al., 2012; Cokley et al., 2013) and research on gender-role orientation in the IP is sparse (September et al., 2001). Our findings indicate that both, female and male students experience impostor feelings but female students experience them more intensely, which aligns to previous findings (King and Cooley, 1995; Kumar and Jagacinski, 2006; McGregor et al., 2008; Jöstl et al., 2012). Moreover, in line with September et al. (2001) findings, students with undifferentiated or feminine gender-role orientation suffer more intensely from the IP than students with masculine or androgynous gender-role orientation. Focusing exclusively on gender without including gender-role orientation might account for inconsistencies in previous findings regarding gender differences in the IP when samples of women high in masculinity or men high in femininity were investigated. Moreover, examining gender and gender-role orientation simultaneously allows for a better understanding of gender differences as interactions between both variables can be investigated. In our study, we found no statistically detectable interactions between gender and gender-role orientation, indicating that gender and gender-role orientation independently contribute to the intensity of the IP. This means that regardless of their gender-role orientation women suffer more from the IP than men, and that feminine and undifferentiated gender-role orientations are equally conducive to the IP for women and men. Gender might account for differences in the IP as high achieving women – regardless of their gender-role orientation – face the dilemma of stereotype incongruence (Eagly and Karau, 2002). High intellectual ability is seen as a feature of men rather than women (Furnham et al., 2002; Bian et al., 2017). If women act in accordance to this social stereotype, they are viewed as incapable; if they do not, they lose their femininity (Eagly and Karau, 2002). Therefore, women might diminish this ambivalence by devaluating their success (Clance and O’Toole, 1987). The contribution of gender-role orientation to the IP can be explained via the concrete characteristics of masculinity and femininity. Individuals with masculine gender-role orientation describe themselves as dominant, ambitious, assertive and competitive, and are convinced that they have leadership abilities. These traits clearly contradict the traits of IP sufferers who do not believe in their competences. Individuals with feminine gender-role orientation, however, describe themselves as understanding, compassionate, gentle, warm, and sensitive to the needs of others. These traits are not linked to competence but to kindness. Individuals who attribute feminine but not masculine characteristics to themselves, might believe that their success is due to these traits rather than due to their competence. Although effect sizes are small, gender-role orientation explains differences in the IP more comprehensively than gender. This further emphasizes the need for a more holistic approach to assessing gender differences in the IP than simply comparing men and women.

Empirical evidence positioning the IP as a potential barrier for academic careers stresses the need for finding ways to overcome the IP. Self-compassion, a construct associated with positive health related, motivational, and emotional aspects (Neff et al., 2005; Barnard and Curry, 2012; Breines and Chen, 2012; MacBeth and Gumley, 2012; Sbarra et al., 2012; Neff and Germer, 2013), was examined as a potential resilience factor against the IP. Our findings support this hypothesis as the intensity of the IP decreases with an increasing level of self-compassion. Moreover, the likelihood to be highly self-compassionate and at the same time experience intense impostor feelings is very small. Thus, interventions tailored to increase self-compassion might be an effective way to support students suffering from the IP in overcoming their impostor feelings. Central components of the IP such as fear of failure, denial of competence, and self-doubts are buffered by self-compassion (Clance, 1985b; Fried-Buchalter, 1997; Thompson et al., 2000; Neff et al., 2005; Neff and McGehee, 2010). The inverse relationship of self-compassion and the IP is also emphasized by associates with other constructs. While students suffering from the IP set themselves unfavorable achievement goals (Kumar and Jagacinski, 2006), self-compassionate students tend to set themselves goals beneficial for academic achievement (Neff et al., 2005). Self-compassion is further associated with increased motivation whereas the IP is theoretically related to drop-out intentions (Topping and Kimmel, 1985). Additionally, depression and anxiety are positively associated with the IP and negatively associated with self-compassion (McGregor et al., 2008; Barnard and Curry, 2012; MacBeth and Gumley, 2012).

Overall, our findings highlight that students who experience the IP lack self-compassion. More specifically, the IP is positively correlated with negative components of self-compassion and negatively associated with positive components of self-compassion. Our findings indicate that IP sufferers lack self-kindness and mindfulness. The IP is associated with positive and negative components of self-compassion but effect sizes are moderate to large for correlations between the IP and negative components and small for correlations between the IP and positive components. Thus, the IP is more strongly associated with negative components than with positive components of self-compassion. In our study, students suffering from the IP are highly self-judgmental, over-identify with negative aspects of themselves or their lives, and isolate themselves from others in terms of believing only they are imperfect and fail. Even though isolation is highly correlated with the IP, we did not find a statistically detectable relationship between the IP and common humanity, the positive counterpart of isolation. Possibly, IP sufferers do agree that failing is part of the human condition (which is the content of the items measuring common humanity) as they are always in fear of failing. However, due to their need to be special and their superman/superwoman aspects, IP sufferers might feel threatened rather than relieved by this fact. Therefore, different mechanisms underlying answering styles of IP sufferers and individuals who do not suffer from the IP might account for this contradictory finding. To clarify this, further research should differentiate between the components of the IP and examine associations between these components and the components of self-compassion on a deeper level. The instrument used to assess the IP in this study, unfortunately does not depict single components of the IP and thus does not allow such systematic investigations.

To enhance understanding about psychological mechanisms contributing to academic attrition of female university students, we examined a model spanning femininity and masculinity, self-compassion, and the IP for all students, and for female and male students separately. Previous research indicates that the IP is positively associated with feminine gender-role orientation and negatively associated with masculine gender-role orientation (September et al., 2001). Self-compassion is positively associated with both femininity and masculinity, whereas the relationship between self-compassion and masculinity is stronger than the relationship between self-compassion and femininity (Tatum, 2013), and negatively associated with the IP. We thus hypothesized that differences in self-compassion partly account for the relationship between gender-role orientation and the IP. Individuals with high femininity might show lower levels of self-compassion that in turn might account for higher levels of the IP. When including all (male and female) students in the model, all direct and indirect effects are statistically detectable, indicating that self-compassion indeed mediates the relationship between gender-role orientation and the IP. Feminine gender-role orientation is positively associated with the IP while masculine gender-role orientation is negatively associated with the IP, which is in line with previous findings (September et al., 2001). Femininity and masculinity are both positively associated with self-compassion with a stronger association for masculinity. This aligns to Tatum (2013) finding that masculinity accounts for the greatest proportion of variance in self-compassion, and to September et al. (2001) findings that masculine students experience greater levels of well-being than feminine students. It is well documented that self-compassion contributes to increased well-being (Allen et al., 2012; Barnard and Curry, 2012; MacBeth and Gumley, 2012; Sbarra et al., 2012). This is also reflected in the finding that self-compassion is highly negatively associated with the IP, which is associated with reduced well-being (September et al., 2001; McGregor et al., 2008; Cokley et al., 2013). Moreover, these findings are in line with our previous findings that students possessing low levels of femininity and masculinity, i.e., undifferentiated students, experience the IP more intensely and are less self-compassionate than androgynous students who possess high levels of both masculinity and femininity. Relationships between gender-role orientation and the IP mediated by self-compassion are both negative. This highlights the resilience function of self-compassion for the IP and indicates that students with feminine and undifferentiated gender-role orientation would benefit most from facilitation of self-compassion in academic contexts. Comparing results for female and male students, it is apparent that the relationship between self-compassion and the IP is stronger for female than male students. This aligns to empirical evidence suggesting that females score lower on measures of self-compassion but higher on measurers of the IP than males (McGregor et al., 2008; Jöstl et al., 2012; Yarnell et al., 2015). Even though self-compassion seems to be a resilience factor against the IP for female and male students, this finding emphasizes that female students would benefit more from interventions designed to increase self-compassion than male students.

### Limitations and Implications for Future Research

Three limitations of this study should be considered. First, our findings are based on self-report measures. It is thus possible that our data is biased due to response biases, e.g., social desirability. However, participation was anonymous and voluntary, and confidentiality of the data was assured. As this study was designed as an online-survey, participants chose a setting in which they felt comfortable answering the questionnaire. We thus assume that response bias is small and balanced by the large sample size. Second, the cross-sectional design of this study allowed us to gain new insights in the prevalence of the IP in first-year undergraduates, to enhance understanding of the leaky pipeline of female academic careers. The design of future studies should be tailored to the process characteristic of the leaky pipeline of female academics over time. A longitudinal design would be appropriate to grasp the nature of this phenomenon more comprehensively. Third, it should be considered that correlational findings do not imply directionality. Consequently, it is not clear if self-compassion is a resilience factor against the IP or if the IP prevents students from developing self-compassion. Thus, future research is needed to further examine our hypothesis that self-compassion is a resilience factor against the IP. A true experiment evaluating the effectiveness of an intervention designed to increase self-compassion for students experiencing different intensity levels of the IP would be an appropriate study design to further examine this research question.

## Conclusion

The present study shows that the IP is highly prevalent in first-year undergraduate students, especially in female students, and in students with feminine and undifferentiated gender-role orientation. It is well documented that the IP is associated with motivational, emotional, and health related aspects that interfere with academic achievement and hinder academic careers (Topping and Kimmel, 1985; Cozzarelli and Major, 1990; Chrisman et al., 1995; Fried-Buchalter, 1997; Thompson et al., 1998, 2000; Leary et al., 2000; Sonnak and Towell, 2001; Kumar and Jagacinski, 2006; Jöstl et al., 2012; Cokley et al., 2015). Moreover, the IP is theoretically related to academic attrition (Topping and Kimmel, 1985). It is thus a promising explanatory factor for academic drop-out of female students and those with feminine or undifferentiated gender-role orientation. This stresses the need for finding ways to support students who are suffering from the IP. Our findings show that self-compassion is a resilience factor against the IP, and that feminine or undifferentiated students would benefit most from facilitation of self-compassion. In recent years, numerous interventions to facilitate self-compassion have been implemented and effectively increased self-compassion, life satisfaction, and decreased depression, anxiety, and stress (Gilbert, 2005; Neff and Germer, 2013). In contrast, the IP was first studied by Clance and Imes (1978) in the clinical field and despite increased empirical attention to the IP, treatment is still limited to psychotherapy (Langford and Clance, 1993) and there is no evidence for its effectiveness in this context. Considering that students suffering from the IP isolate themselves from others, are trying to hide failure, are constantly on the watch not to get unmasked as unintelligent, while expecting themselves to master every challenge on their own, it seems reasonable to assume that these students might have internal barriers to psychotherapy. Interventions of self-compassion are less stigmatizing and might thus be more approachable for individuals suffering from the IP. Therefore, facilitating self-compassion in IP sufferers to help them overcome their impostor feelings seems to be a promising way to patch the leaky pipeline of female academic careers.

## Ethics Statement

This study was carried out in compliance with ethical standards of the Austrian Federal Ministry of Health (Bundesministerium für Gesundheit, 1995) and the American Psychological Association (American Psychological Association, 2010). Prior to participation, students gave written informed consent. The consent form informed participants about duration, procedure, and goals of this study. Participants were guaranteed anonymity and confidentiality of their data and were informed that participation was voluntary and could be withdrawn at any point of the questionnaire. According to Austrian and European (EU) law, approval of an ethics committee was not necessary as this study did not involve patients, was non-invasive, and participation was voluntary and anonymous. There is no institutional review board (IRB) at the University of Vienna, where this research was conducted. Hence, no IRB approval was necessary.

## Author Contributions

All listed authors contributed meaningfully to the paper. AP and MK developed the study concept. All authors contributed to the study design, analyzed or interpreted the data. AP and MK prepared the draft manuscript and BS provided critical revisions. All authors approved the final version of the manuscript for submission.

## Conflict of Interest Statement

The authors declare that the research was conducted in the absence of any commercial or financial relationships that could be construed as a potential conflict of interest.
